# Prevalence of colistin-resistant *Escherichia coli* in foods and food-producing animals through the food chain: A worldwide systematic review and meta-analysis

**DOI:** 10.1016/j.heliyon.2024.e26579

**Published:** 2024-02-22

**Authors:** Florencia Aylen Lencina, Matías Bertona, María Angeles Stegmayer, Carolina Raquel Olivero, Laureano Sebastián Frizzo, Jorge Alberto Zimmermann, Marcelo Lisandro Signorini, Lorena Paola Soto, María Virginia Zbrun

**Affiliations:** a Laboratory of Food Analysis, Institute of Veterinary Science (ICiVet Litoral), National University of the Litoral, National Council of Scientific and Technical Research (UNL/CONICET), Esperanza, Argentina; b Department of Public Health, Faculty of Veterinary Science – Litoral National University, Esperanza, Argentina; c Instituto de Investigación de la Cadena Láctea (INTA-CONICET), Estación Experimental Agropecuaria Rafaela, Ruta 34 Km 227, Rafaela, Santa Fe, Argentina

**Keywords:** Colistin resistance, *Escherichia coli*, Foods and food-producing animals, Meta-analysis, Prevalence, *mcr* gene

## Abstract

The purpose of this systematic review and meta-analysis was to summarize the available scientific evidence on the prevalence of colistin-resistant *Escherichia coli* strains isolated from foods and food-producing animals, the mobile colistin-resistant genes involved, and the impact of the associated variables. A systematic review was carried out in databases according to selection criteria and search strategies established *a priori*. Random‐effect meta‐analysis models were fitted to estimate the prevalence of colistin-resistant *Escherichia coli* and to identify the factors associated with the outcome. In general, 4.79% (95% CI: 3.98%–5.76%) of the food and food-producing animal samples harbored colistin-resistant *Escherichia coli (total number of colistin-resistant Escherichia coli/total number of samples)*, while 5.70% (95% confidence interval: 4.97%–6.52%) of the *E. coli* strains isolated from food and food-producing animal samples harbored colistin resistance (total number of colistin-resistant *Escherichia coli*/total number of *Escherichia coli* isolated samples). The prevalence of colistin-resistant *Escherichia coli* increased over time (*P* < 0.001). On the other hand, 65.30% (95% confidence interval: 57.77%–72.14%) of colistin resistance was mediated by the *mobile colistin resistance-1* gene. The *mobile colistin resistance-1* gene prevalence did not show increases over time (*P* = 0.640). According to the findings, other allelic variants (*mobile colistin resistance 2–10* genes) seem to have less impact on prevalence. A higher prevalence of colistin resistance was estimated in developing countries (*P* < 0.001), especially in samples (feces and intestinal content, meat, and viscera) derived from poultry and pigs (*P* < 0.001). The *mobile colistin resistance-1* gene showed a global distribution with a high prevalence in most of the regions analyzed (>50%). The prevalence of colistin-resistant *Escherichia coli* and the *mobile colistin resistance-1* gene has a strong impact on the entire food chain. The high prevalence estimated in the retail market represents a potential risk for consumers' health. There is an urgent need to implement based-evidence risk management measures under the “One Health” approach to guarantee public health, food safety, and a sustainable future.

## Highlights

• Wide spread of colistin-resistant *E. coli* was demonstrated in foods and in the main points of the food chain.
• The *mcr-1* gene is the primary plasmid mechanism involved in colistin resistance in foods.
• Developing countries and poultry and pigs have higher levels of colistin resistance.
• The results suggest the potential spread of colistin-resistant *E. coli* from “farm to table”.

## 1 Introduction

Antimicrobial resistance (AMR) has become one of the biggest threats to global health, development, and food safety [1]. Although AMR represents a natural aspect of bacterial evolution, this process has accelerated and expanded due, among other factors, to antimicrobials use in human and veterinary medicine [2,3]. Currently, at least 700,000 people die each year due to drug-resistant diseases, and this scenario could rise to 10 million deaths every year by 2050 if no additional measures are taken [4,5].

*Escherichia coli* is the predominant microorganism in the gastrointestinal tract of humans and warm-blooded animals, being a common foodborne pathogen of humans worldwide [6]. Most *E. coli* strains are commensal; however, some strains cause gastrointestinal infections while others cause urinary, nervous, and systemic infections [7,8]. Furthermore, *Escherichia coli* is typically chosen as a representative Gram-negative indicator of AMR because of its ability to harbor several resistance determinants and disseminate resistance to potentially pathogenic or zoonotic bacteria [9,10]. Thus, the use of this bacteria makes it feasible to compare levels of resistance between populations [10]. Extended-spectrum beta-lactamase-producing Enterobacterias, such as *E. coli*, have been increasingly associated with serious public health problems due to limited treatment options [11]. In this context, the use of antibiotics of last resort, such as colistin, has increased [12].

Colistin (polymyxin E) is a last-resource antimicrobial (ATM) used for the human potentially fatal infection treatment by multiresistant-enterobacteria [2,12]. The WHO classified polymyxins in the group of “Highest Priority Critically Important Antimicrobials for human medicine” [13]. In addition, colistin has been continuously used in livestock production for prophylactic, therapeutic, and even for growth promotion purposes [14,15]. However, the use of colistin has already been limited by several regulations due to the emergence of resistant bacteria to this polymixin [11,[16], [17], [18], [19], [20], [21], [22], [23], [24], [25], [26], [27], [28]].

The mechanisms involved in colistin resistance are due to chromosomal mutations or the acquisition of transferable plasmids that carry the genes of the *mobile colistin resistance* (*mcr)* family [29]. Chromosomal alterations are related to changes in one or several genes (e.g. *pmrA*, *pmrB*, *mgrB*, *phoP*, and *phoQ* genes in *E. coli* isolated from livestock) that participate in the biosynthesis of lipid A of the outer bacterial membrane [[30], [31], [32]]. This mechanism is capable of being transferred vertically from generation to generation [33]. In contrast, plasmid-mediated colistin resistance has a potential implication for the horizontal movement of resistance among different bacterial species or bacteria with different virulence profiles (e.g. from bacteria commensals to pathogenic bacteria) [30,34]. In the plasmid-mediated and transmissible colistin resistance, the *mcr-1* gene encodes a phosphoethanolamine transferase that incorporates a phosphoethanolamine group to lipid A [30,35]. In both mechanisms, the negative charges of the lipopolysaccharide are affected (decreased), thus decreasing interaction with the positively charged polymyxins [30,35]. Although the *mcr-1* gene has been detected mainly in *E. coli*, other *Enterobacteriaceae* genera, such as *Salmonella*, *Shigella*, *Klebsiella* and *Enterobacter* carry this gene [36]. In this sense, the *mcr-1* gene has been reported in various plasmid backbones (IncI2, IncHI 2, IncP, IncX4, IncFI, and IncFIB) [29,35,37,38], all of which are known to be vehicles disseminating antibiotic resistance genes among *Enterobacteriaceae*. The *mcr-1* gene could also be mobilized with other mobile genetic elements like transposons and integrons carrying multidrug resistance determinants [39]. Finally, to date, an additional eight distinct mobile colistin-resistant genes, *mcr*-2 to *mcr*-*10*, have been identified [[40], [41], [42], [43], [44], [45], [46], [47], [48]].

Of great current concern is the possible transmission of colistin-resistant *E. coli* (regardless of the mechanism involved) to humans through the food chain due to the possibility of causing difficult-to-treat diseases if the conditions are given (pathogenic strains, adequate infective doses, etc.) [49,50]. In addition, if these bacteria carry mobile colistin resistance mechanisms, they can eventually colonize the human microbiota and horizontally transmit resistance to potentially pathogenic enterobacteria [34]. In this context, considerable worldwide literature has been published about the prevalence of colistin-resistant *E. coli* and its resistance mechanisms in strains isolated from food and food-producing animals.

One of the strategic objectives of the WHO Global Action Plan for AMR focuses on strengthening knowledge and the scientific basis through research on AMR that supports measures and investments to address them [51]. In this sense, the aim of this study was to synthesize and integrate the available information on the current global situation regarding food and the food chain. A systematic review and meta-analysis were performed to describe the epidemiology of colistin resistance in *E. coli* strains isolated from foods and food-producing animals from “farm to table”. This study integrated existing scientific papers to estimate the prevalence of colistin-resistant *E. coli* (regardless of the mechanism involved). Afterwards, when it became possible, its association with the *mcr* genes involved was estimated. This information is intended to serve for the development of evidence-based risk management measures under the “One Health” approach to guarantee high standards of food safety, consumer and population health, and to avoid higher costs in the future.

## 2 Materials and methods

This systematic review and meta-analysis was performed in accordance with the Preferred Reporting Items for Systematic Reviews and Meta-Analyses (PRISMA) guidelines [52]. The working protocol was performed in five steps: search strategy, study selection, outcome variable, information extraction, and statistical analysis.

### 2.1 Search strategy

Relevant worldwide literature published between 1973 to April 2021 was identified from the Scielo, Pubmed, ScienceDirect, Dialnet, and Scopus databases. The following specific search terms were used: *colistin, resistance*, *Escherichia coli*, and *foods*. Also, specific boolean terms “and”/“or” were used. The suitability of the scientific papers based on their titles and abstracts was analyzed in agreement with the inclusion/exclusion criteria. Only scientific papers written in English, Spanish, Portuguese, or French were included. Potentially useful articles obtained from the database search strategy used were imported into the Mendeley Desktop reference management software version 1.19.5 (Mendeley Ltd., Elsevier, Netherlands). Duplicate records were identified, documented, and removed with Mendeley. Some duplicates were addressed manually due to variation in reference styles across sources.

### 2.2 Study selection

Two researchers selected the first group of suitable scientific papers, independently. They resolved any disagreement by consensus or by the intervention of a third researcher. The selection of the scientific papers included in this meta-analysis was based on the following criteria: they should be cross-sectional observational prevalence or incidence studies published in peer-reviewed journals, without published time restriction (and exceptionally, degree theses and short communications), which analyzed food and food-producing animals. The scientific papers must have reported the total number of samples analyzed and/or the number of *E. coli* strains isolated and the total number of *E. coli* phenotypically resistant to colistin. When the identification of the total number of resistant *E. coli* carrying *mcr* genes was available, this information was included in the analysis. Scientific papers that did not have the above criteria, duplicate reports, reviews, and scientific papers without access to the full text (after e-mail communication with the authors) were excluded.

We searched related reviews and references for relevant scientific papers, which facilitated the location of those scientific papers not indexed by the proposed keywords. These papers were denominated “articles identified through other sources”. In addition, we contacted expert authors of scientific papers by e-mail when we could not access the full document in order to request the data for inclusion in this meta-analysis.

### 2.3 Definition of the outcome variable

We established three types of prevalence analyses: A, B, and C. This was done to include as many publications as possible and to evaluate trends according to the relationships used. The prevalence of colistin-resistant *E. coli* was calculated in Analysis A from the total number of colistin-resistant *E. coli* isolated over the total number of samples. In Analysis B, the prevalence was calculated from the total number of colistin-resistant *E. coli* isolated over the total number of *E. coli* isolated. In Analysis C, the prevalence of the *mcr* gene was calculated from the total number of *mcr* genes detected over the total number of colistin-resistant *E. coli* isolated (this analysis was performed for each *mcr* gene: *1–10*).

### 2.4 Information extraction

The same two researchers individually extracted the data from the selected scientific papers using Microsoft Excel. After fully reading each document selected in the previous stage, we extracted qualitative information about the geographic distribution (South America, North America, Africa, Europe, Asia), sampling point (farm, slaughterhouse, food market), animal species or plant-based products analyzed (animal species: bovines, pigs, poultry, aquatic species, reptiles, other food-producing mammals; plant-based food products: vegetables and fruit), type of sample (feces and intestinal contents, meat/carcasses, viscera, eggs, milk, and dairy products). For the “type of sample”, we considered the feces and intestinal contents of food-producing animals due to the relationship of these samples with food cross-contamination along the food chain [53]. In addition, the following quantitative information was extracted: sampling year, sample size, number of *E. coli* colonies isolated, number of colistin-resistant *E. coli,* and number of the *mcr* gene (*1–10*) carriage. When a scientific paper reported the results derived from different conditions (i.e., region of origin, prevalence estimation in different years, different species or samples, etc.), each condition was considered as an individual outcome, named “prevalence report”. Therefore, each scientific article could contain more than one prevalence report. When the sampling year was not available, the publication year was used. No scores were used to exclude scientific papers [54].

### 2.5 Statistical analysis

All statistical analyses were performed using Comprehensive Meta‐Analysis software version 2.2 [55]. A random-effects model was employed. In each analysis, the determination of the heterogeneity of scientific papers was carried out using the *I*^*2*^ test to assess the appropriateness of pooling data [56].

A series of sub-analyses were performed in order to assess the impact of the different categories of variables on the prevalence of colistin-resistant *E. coli* and the *mcr-1* gene, and to analyze possible sources of heterogeneity. For the Analysis C of the *mcr 2–10* genes, only a global prevalence analysis was performed.

On the other hand, a meta-regression analysis was performed to explore the sources of heterogeneity by evaluating the relationship between the year analyzed in each publication and the prevalence of colistin-resistant *E. coli* (analyses A and B) and *mcr-1* genes (analysis C). For this subgroup, we considered the year of sampling. If this information was not available, we considered the year of publication instead of the sampling year since the year of publication of a scientific article is usually close (two or three years) to the year in which the study was conducted.

An adjusted rank correlation test using the Egger's method [57] and Begg's test [58] was used to assess publication bias. To calculate the number of scientific papers that would have been needed to reverse the effect, the *trim and fill* process was used.

The pooled prevalence estimate (*p*-estimate) were expressed as percentages with a 95% confidence interval (CI). The statistical significance used for both the meta-analysis models and the publication bias tests was *P* < 0.05.

## 3 Results

### 3.1 Excluded articles

**Fig. 1:**
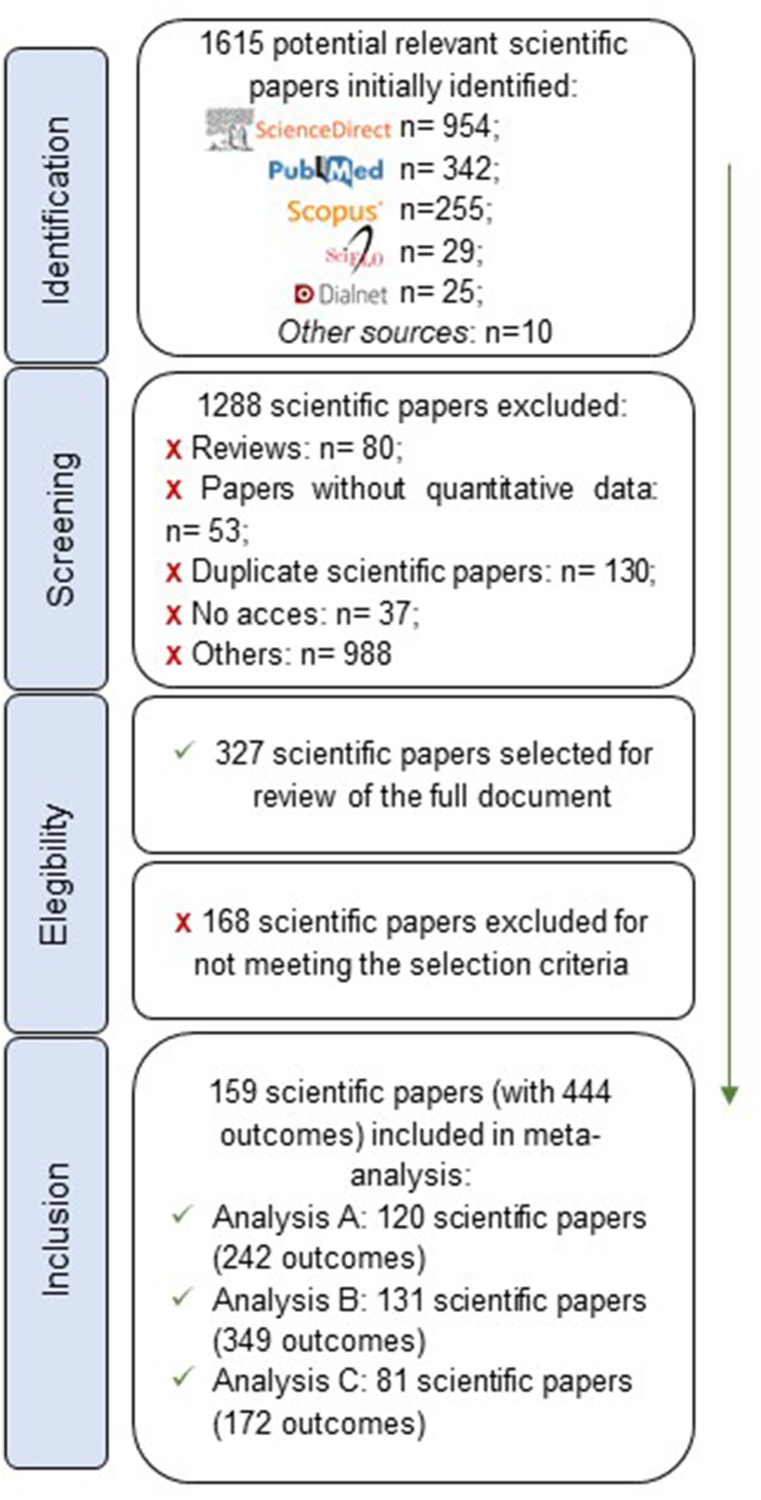
Flow diagram (according to PRISMA guides) of selected studies included in the meta‐analysis. Abbreviation: n: Number of reports included and analyzed.

A total of 1615 scientific papers were identified in the databases and through other sources. After reading the title and abstract in the first stage (screening), and the import to Mendeley Desktop reference management software, 1288 scientific papers were excluded. Furthermore, after reading the full text in the second stage (selection), 168 scientific papers were excluded (Fig. 1).

### 3.2 Overview of included articles

Four hundred forty-four prevalence reports from 159 scientific papers were included in the meta-analysis. According to the data provided by each report, they were included in the different analyses (A, B, and/or C) (Fig. 1).

From the 444 prevalence reports included, six (1.35%) were published between 1973 and 1990, four (0.90%) between 1991 and 2000, forty-two (9.46%) between 2001 and 2010, and forty-nine (11.04%) between 2011 and 2015. The majority of reports (n = 343, 77.25%) were published from 2016 to 2021. The location of reports covered more than 50 different countries corresponding to five geographical regions for Analysis A and Analysis B (except Antarctica), with the majority of outcomes from Asia (mainly China) with 166 reports, and European countries (mainly Germany) with 180 reports. For Analysis C, the presence of the *mcr* genes was evaluated in 37 countries corresponding to four geographical regions (except Antarctica). In Oceania, only one report was detected; therefore, this region was not included in the analyses due to its low representativeness.

### 3.3 Global prevalence of colistin-resistant *E. coli*

The pooled prevalence estimate of colistin-resistant *E. coli* was similar for Analysis A and for Analysis B (Table 1). On the other hand, the *p*-estimate in Analysis C was high for the *mcr-1* gene, while it was relatively lower and variable for other *mcr* genes (Table 1). High heterogeneity was observed across the reports included in the analyses A, B, and C for the *mcr-1* gene. Variable heterogeneity was observed for the *mcr 2–10* genes (Table 1).

**Table 1:** Summary of global prevalence, heterogeneity and number of reports in the A, B and C analyses.

Analysis		*p*-estimate (%)	95% CI	I^2^ (%)	Number of reports
A		4.79	3.98–5.56	96.84	242
B		5.70	4.97–6.52	96.89	349
C	*mcr-1*	65.30	57.78–72.14	92.59	172
	*mcr-2*	4.24	2.76–6.45	78.86	79
	*mcr-3*	5.14	3.43–7.62	41.31	60
	*mcr-4*	5.34	2.73–10.17	80.02	47
	*mcr-5*	6.12	4.36–8.52	0	42
	*mcr-6*	5.65	3.49–9.01	0	26
	*mcr-7*	5.65	3.49–9.01	0	26
	*mcr-8*	4.32	2.56–7.20	0	26
	*mcr-9*	7.29	1.59–27.66	78.77	11
	*mcr-10*	6.68	1.33–27.57	0	3

### 3.4 Evolution of colistin-resistant *E. coli* and *mcr-1* gene prevalence throughout the period analyzed

The meta‐regression analysis showed that the prevalence of colistin-resistant *E. coli* in foods and food-producing animals has increased over the years for Analysis A (*P* < 0.001). The same ascending pattern was observed in Analysis B (*P* < 0.001), with a slightly higher slope, compared to Analysis A. However, these analyses did not show any evidence that the *mcr-1* gene prevalence in colistin-resistant *E. coli* shifted over time (*P* = 0.640) (Table 2).

**Table 2:** Summary of random weighted meta‐regression analysis for sampling year as the independent variable, and the prevalence of colistin-resistant *E. coli* (Analysis A and B) and mcr-1 gene (Analysis C) isolates from food and food-producing animals as the outcome variable.

Analysis	Intercept	Slope	*p*-value
A	−111.57	0.0539	<0.001
B	−112.56	0.0545	<0.001
C	51.82	−0.0254	0.6388

### 3.5 Prevalence of colistin-resistant *E. coli* and *mcr-1* gene across geographic regions

**Fig. 2:**
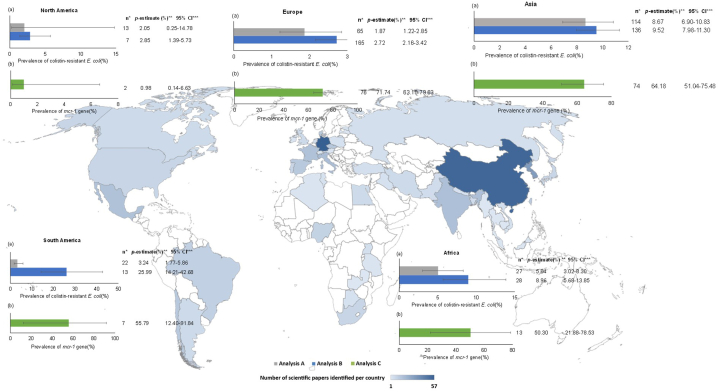
Bar graph: Subgroup analysis comparing the prevalence of colistin-resistant *E. coli* (a) and *mcr-1* gene (b) in food and food‐producing animals across geographic regions. World map graph: Geographical distribution (by country) of scientific papers included. The intensity of the blue color is proportional to the number of studies detected in each country. In countries without color, no studies were detected or included in this meta-analysis. **References**: The horizontal bars extend to the pooled prevalence estimate in each geographic region. The horizontal lines indicate confidence intervals. Abbreviations: *Number of reports included and analyzed, **Pooled prevalence estimate, ***Confidence interval.

Studies conducted in Asia, Africa, and Latin America showed the highest prevalence of colistin-resistant *E. coli*, while the lowest prevalence was detected in Europe and North America (*P* < 0.001; Fig. 2 (a)). The *p*-estimates were higher for Analysis B (calculated as the number of colistin-resistant *E. coli* isolated over the total number of *E. coli* isolated) than for Analysis A (calculated as the number of colistin-resistant *E. coli* isolated over the total number of samples) for the majority of geographic regions. Moreover, in Latin America, the highest prevalence was detected in this analysis (Analysis B) (*P* < 0.001; Fig. 2 (a)).

On the other hand, studies conducted in all geographic regions demonstrated a high prevalence of the *mcr-1* gene in colistin-resistant *E. coli*, except in North America (*P* < 0.001; Fig. 2 (b)).

### 3.6 Prevalence of colistin-resistant *E. coli* and *mcr-1* gene considering the site of sampling

**Fig. 3:**
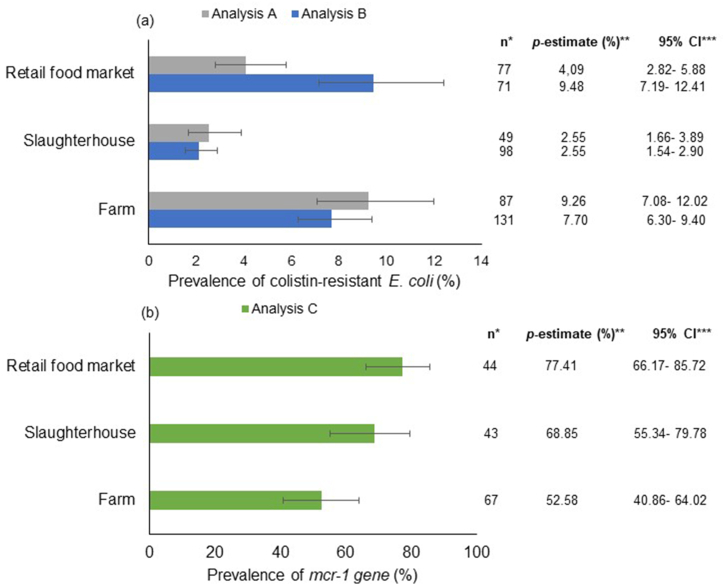
Subgroup analysis comparing the prevalence of colistin-resistant *E. coli* (a) and *mcr-1* gene (b) in food and food‐producing animals considering the site of sampling. **References**: The horizontal bars extend to the pooled prevalence estimate in each geographic region. The horizontal lines indicate confidence intervals. Abbreviations: *Number of reports included and analyzed, **Pooled prevalence estimate, ***Confidence interval.

Primarily, food and food-producing animals were sampled directly at farms; however, samples in slaughterhouses and food markets were also taken. The *p*-estimate of colistin-resistant *E. coli* at farm was higher than the estimate in slaughterhouses for Analysis A and Analysis B (*P* < 0.001). Regarding food markets, the *p*-estimate for Analysis A was lower than that detected at farm (*P* < 0.001) but similar to the one estimated in the slaughterhouse. However, food markets for Analysis B showed the highest prevalence of colistin-resistant *E. coli*, but they were not different when compared with the prevalence estimated at farm. In this analysis, the slaughterhouse showed the lowest *p*-estimate of colistin-resistant *E. coli* (*P* < 0.001) (Fig. 3 (a)).

The *mcr-1* gene prevalence among colistin-resistant *E. coli* in the food market was higher than that estimated at farm (*P* = 0.0236). However, the *p*-estimate in the slaughterhouse was not different compared with the prevalence estimated in the rest of the sites, according to Analysis C (Fig. 3 (b)).

### 3.7 Prevalence of colistin-resistant *E. coli* and *mcr-1* gene in foods: animal species and plant-based products

**Fig. 4:**
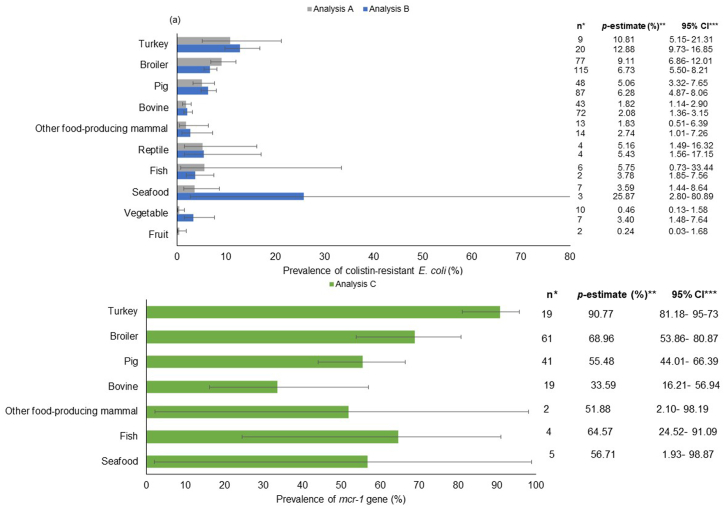
Subgroup analysis comparing the prevalence of colistin-resistant *E. coli* (a) and *mcr-1* gene (b) in food and food‐producing animals according to the species/plant-based product analyzed. **References**: The horizontal bars extend to the pooled prevalence estimate in each geographic region. The horizontal lines indicate confidence intervals. Abbreviation: *Number of reports included and analyzed, **Pooled prevalence estimate, ***Confidence interval.

Samples taken from turkeys, broilers, and pigs showed the most important prevalence of colistin-resistant *E. coli* in all of the analyses carried out. In contrast, bovines showed the lowest *p*-estimate for Analysis A and B (*P* < 0.001; Fig. 4 (a)). The prevalence of the *mcr-1* gene among colistin-resistant *E. coli* was higher in samples from turkey than that estimated in broiler, pig, and bovine samples (*P* < 0.001; Fig. 4 (b)).

Other food-producing mammals (such as sheep, deer, goats, and rabbits, among others), fish and seafood, and reptiles showed a variable prevalence. However, they were not different compared with the prevalence estimated in the other food-producing animals (Fig. 4 (a)). In addition, the prevalence of the *mcr-1* gene in fish, seafood, and in food-producing mammals did not show differences to the prevalence estimated in the rest of the subgroups analyzed. However, these values are less representative due to the lower number of reports included for these species. For reptiles, it was not possible to obtain enough data to estimate the prevalence of the *mcr-1* gene.

Plant-based products such as fruits and vegetables showed a different prevalence according to the analyses carried out. Vegetables and fruits showed a lower prevalence than broilers and pigs for Analysis A (*P* < 0.001). However, these products did not show differences with the *p*-estimate in the rest of the species (Fig. 4(a)). In contrast, the *p*-estimate for Analysis B in vegetables was only lower than that detected in turkey (*P* < 0.001), while it was not different compared with the prevalence estimated in the other species. As regards fruits, the number of scientific papers included in the meta-analysis was not high enough to estimate the prevalence in Analysis B. Finally, for the plant-based products, it was not possible to obtain enough data to estimate the prevalence of the *mcr-1* gene.

### 3.8 Prevalence of colistin-resistant *E. coli* and *mcr-1* gene considering the type of sample

The matrices sampled to a greater extent were those derived from species of animal origin. The majority of the reports analyzed samples taken from feces and intestinal contents, and from meat (which includes raw or cooked meat, carcasses, and skin). A smaller number of reports analyzed samples of viscera, milk and dairy products, and eggs.

**Fig. 5:**
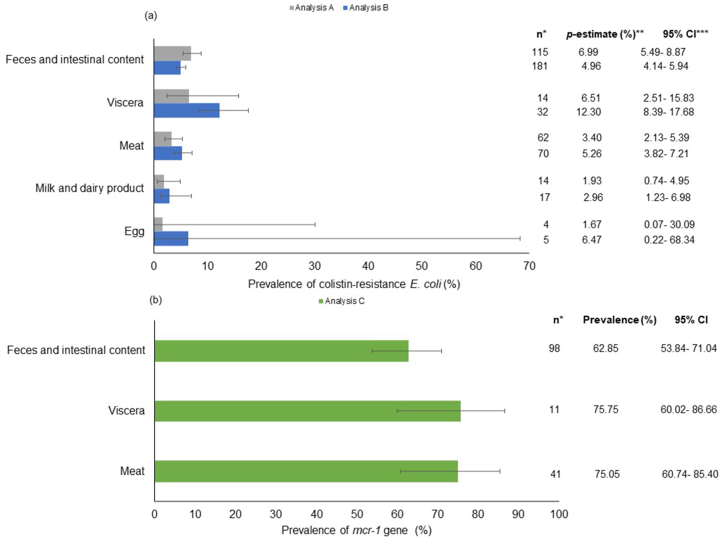
Subgroup analysis comparing the prevalence of colistin-resistant *E. coli* (a) and *mcr-1* gene (b) in food and food‐producing animals according to type of sample analyzed. **References**: The horizontal bars extend to the pooled prevalence estimate in each geographic region. The horizontal lines indicate confidence intervals. Abbreviation: *Number of reports included and analyzed, **Pooled prevalence estimate, ***Confidence interval.

For this subgroup, the results obtained were different according to the prevalence analyses carried out. For Analysis A, feces and intestinal contents samples showed a higher prevalence than that estimated in samples from meat, and milk and dairy products (*P* < 0.001), while they were not different to the *p*-estimate in samples from viscera (Fig. 5 (a)). For Analysis B, the *p*-estimate of colistin-resistant *E. coli* in samples from viscera was higher than the prevalence estimated in samples from feces and intestinal contents, meat, and milk and dairy products (which showed a similar prevalence) (*P* < 0.001) (Fig. 5 (a)).

The prevalence estimated in the samples from eggs did not show differences to the *p*-estimate in the rest of the samples analyzed (Fig. 5 (a)). However, these estimates are less representative due to the lower number of reports included in this sample.

For Analysis C, the prevalence of the *mcr-1* gene revealed differences between the subgroups analyzed (*P* = 0.02). The existence of differences is due to variables with a low number of reports analyzed or variables reported in a combined manner (variables not shown). On the other hand, the prevalence of the *mcr-1* gene estimate in feces and intestinal contents, viscera, and meat did not show differences, being high in all cases (Fig. 5 (b)).

### 3.9 Publication bias

The Egger's test, Begg's test and *Trim and fill* process were used to detect publication bias in the included scientific papers. There was a general tendency of publication bias for analyses B and C; the addition of 32 independent scientific papers was required in Analysis C to reverse the effects (Table 3). However, the large number of scientific papers included in this meta‐analysis provides valid results beyond the potential bias.

**Table 3:** Results of publication bias detection.

**Analysis**	***Trim and fill***	Begg's test (*P*- value)	*Egger's test*	
			Intercept	*P*-value
**A**	0	0.12717	−3.80191	0.00001
**B**	0	0.00008	−2.82574	0.00001
**C**	32	0.04317	1.15182	0.00421

## 4 Discussion

The prevalence of colistin-resistant *E. coli* strains isolated from foods and food-producing animals from 1973 to 2021 and its association with the *mcr* genes were assessed in this meta-analysis. To our knowledge, the present study is the first comprehensive systematic review and meta-analysis of the prevalence of colistin-resistant *E. coli* and *mcr* genes in different food sources along the main points of the food chain.

The analysis of the available information in databases indicates that 4.79% (95% CI: 3.98%–5.76%) of the food and food-producing animal samples were contaminated with colistin-resistant *E. coli* (Analysis A), and that 5.70% (95% CI: 4.97%–6.52%) of the *E. coli* isolated from food and food-producing animal samples presented colistin resistance (Analysis B). In addition, 65.30% (95% CI: 57.77%–72.14%) of these colistin-resistant enterobacteria presented plasmid resistance mechanisms mediated mainly by the *mcr-1* gene. These results are relevant since they offer an updated and integrated global panorama on the role of foods and food-producing animals in the potential transmission and spread of colistin-resistant *E. coli*.

According to the findings, the prevalence of colistin-resistant *E. coli* is relatively low. However, this is not a minor issue since the existence of antimicrobial-resistant bacteria in food is addressed as existing either as a direct hazard or as an indirect hazard for the consumer [34]. The direct hazard (regardless of the resistance mechanisms involved) is related to the potential presence on food of colistin-resistant pathogenic *E. coli*, which can eventually infect a human being after food ingestion or poor food manipulation practices [34]. In this sense, studies suggest an epidemiological relationship between *E. coli*, carriers of virulence genes and *mcr-1* genes isolated from food, humans, and animals, suggesting potential exposure to these bacteria via contaminated food [38,50]. The indirect danger arises through the existence of resistance genes that can be transferred horizontally between commensal bacteria and the main pathogens in the human microbiome when conditions are given [34,59,60]. Although this study confirms the *mcr-1* gene as the predominant variant, *mcr* homologs also have an impact on the presence and spread of colistin resistance. In addition, not all studies that have evaluated colistin resistance have determined the presence of all the reported variants of the *mcr* genes [61]. This could be the reason why the other variants of the *mcr* genes (*2–10*) have been less frequently reported, as well as the reason for the lowest prevalence estimated in this study [62,63].

Since the rise of global concern around the existence and spread of colistin-resistant bacteria mediated by *mcr* genes, several countries have urgently developed and implemented policies and regulations on the use of colistin, particularly in food-producing animals. In this sense, many countries have approved the withdrawal of colistin as a feed additive in animals [16,17,[19], [20], [21], [22], [23], [24],^27^,^28^]. However, in some countries of South America and Africa, colistin is still used as a growth promoter in farm animals, being in many cases an over-the-counter drug [64,65]. Finally, in the European Union, colistin was banned as a growth promoter in food animals (2006), while in Canada and the United States, colistin has never been approved or marketed for food-producing animals [11,18,25,26,66]. In this sense, several studies have sequentially reported a reduction in colistin-resistant *E. coli* and/or the *mcr-1* positive *E. coli* in food animals through the food chain after the colistin ban [19,[67], [68], [69]]. However, the decrease in prevalence was not evident in the global analysis carried out here. According to our findings, the prevalence of colistin-resistant *E. coli* increased over time while the prevalence of the *mcr-1* gene did not show any change over time. Despite regulatory policies, in several countries (e.g. China) colistin is allowed by therapeutic prescription and for prophylaxis or metaphylaxis in livestock, thus resulting in moderate selection pressure on bacteria of animal origin. Furthermore, the rate of resistance reversibility is usually slow, which may justify the patterns observed in this meta-analysis [70]. It is no less important to consider that after the discovery of the *mcr-1* gene, the studies focused on detecting the presence of colistin-resistant *E. coli* and its genetic determinants increased rapidly. In fact, 70.25% of the studies analyzed here were published from 2016 to April 2021. This could also have determined (at least in part) that this global analysis did not detect a decrease in prevalence over time. Therefore, the effect of the new regulations on the levels of resistance to colistin under the “One Health approach" at a global level, as well as the long-term effects, should be further evaluated in the near future.

The highest prevalence of colistin-resistant *E. coli* was observed in Asia, Africa, and especially Latin America, where approximately a quarter of the isolates were resistant. On the other hand, a lower prevalence was detected in countries of Europe and North America. It is suggested that colistin resistance represents a problem that has been extended to a global level, being more common in developing countries. In line with the discussion in the previous paragraph, from the year 2016 many Asian countries as China (2016), Thailand (2017), Japan (2018), Vietnam (2018), Indonesia (2019), Malaysia (2019), and India (2019) have implemented policies and regulations on the use of colistin in livestock [16,17,[19], [20], [21], [22], [23], [24]]. The same happened in many South American countries such as Brazil (2016) and Argentina (2019), among others, and African countries such as South Africa (2016) [27,28,71]. However, the banning of colistin is recent and continues to be used as a prophylactic, metaphylactic or/and preventative treatment in some countries (e.g. China) [19]. In addition, there are still countries in these regions that have not yet taken measures to regulate the use of colistin in farm animals [64,65]. Furthermore, in these countries, several inadequate practices, such as the poor control and monitoring systems for the use of ATMs and containment measures in livestock, as well as the limited health and biosecurity in production plants and waste management, could explain these results [[72], [73], [74], [75], [76]]. The intensification in food production and the minimization of the public health and food security problem of AMR in the food chain complicate this scenario. Moreover, the export of these foods favors the propagation of these resistant enterobacteria throughout the world, even to countries with rigorous control systems [75,77,78]. In contrast, the ban on the use of colistin as a growth promoter in animals in the EU (since 2006) and the not-approved colistin usage in animal feed in the USA and Canada were early [18,25,26]. This could explain (at least in part) why the prevalence of colistin resistance in *E. coli* is low in Europe and very low in North America.

The present study revealed a wide dispersion of the *mcr-1* gene. The high *p*-estimate of the *mcr-1* gene in Europe is probably due to its greater research and documentation over time due to active surveillance programs. On the other hand, in North America, colistin-resistant *E. coli* were still isolated, and the presence of the *mcr-1* gene in some of these resistant bacteria was demonstrated. This shows the global spread of colistin-resistant bacteria, even to regions where its use is not authorized in food animals. Finally, although indicative, the prevalence detected in the analyses carried out for North America should be interpreted with caution. The low number of reports detected and included is partly due to the absence of colistin use in food animals in this region, and partly because some studies did not provide the data required to be included in this meta-analysis.

Concerning the main stages of the food chain and the type of sample analyzed, our results strongly suggest the potential spread of colistin-resistant *E. coli* and the *mcr-1* gene from “farm to table”. Food-producing animals at farm could be considered the original source and reservoir of colistin-resistant *E. coli* due to the high frequency, for decades, of the use of this ATM in livestock [53,79]. Due to the selection pressure imposed by the colistin used in the farm, the fecal microbiota from food-producing animals contains an important proportion of resistant bacteria [10,35]. The contact between plants, water, aquatic species or wild species and food-producing animals’ waste (through cross-contamination, use of fertilizers, irrigation, integrated farming systems, or environmental pollution) may contribute to the animal, food, and environment dissemination of AMR [65,80,81,82]. Moreover, colistin-resistant *E. coli* from the gastrointestinal tract of food-producing animals (intestinal content) can favor cross-contamination of carcasses during slaughter and processing [53,80]. Cross-contamination with antimicrobial-resistant bacteria and/or antimicrobial-resistant genes can spread to other foods during handling in the last stages of the food chain [80]. This could explain the considerable prevalence of colistin-resistant *E. coli,* particularly mediated by plasmidic determinants (*mcr-1*) detected in the food market, as well as the considerable prevalence estimated in meat and viscera samples.

According to our findings, eggs, and milk and dairy products could also be potential sources of colistin-resistant *E. coli*. However, more evidence is needed to determine the relative risk for human health and food safety represented by these foods, especially eggs, as well as the abundance of the *mcr-1* gene in the colistin-resistant *E. coli* isolates.

Another remarkable point is related to the higher prevalence of colistin-resistant *E. coli* found in turkeys, broilers, and pigs. It can be assumed that these findings are a reflection of the intensive animal production system observed mainly in broilers and pigs. Considering the countries that are large producers of food-producing animals, such as China and Brazil, the chicken and pig husbandry industries account for over 96% of total colistin sulfate usage (chicken: 49.01%; pig: 47.41%), according to a report published in 2019 [19,83,84]. Consequently, the selection pressure imposed by the use of colistin is likely to be huge in these animal husbandries. In this sense, according to a review by Binsker et al. [11], a strong positive correlation was revealed between the resistance to and the consumption of colistin in food-producing animals.

This scenario is worrying, considering that poultry and pig meat is positioned as the preferred choice for consumers, especially in developing countries [85]. In contrast, as regards bovines, colistin is used to a lesser extent and has never been approved for use in China [19,84], which may probably explain the lower prevalence of resistant and mobile genes in this species.

Scientific papers on the prevalence of colistin-resistant *E. coli* are still scarce and fragmentary for certain foods. However, our findings suggest that aquatic species (fish and seafood), other food-producing mammals, and reptiles are also contaminated with colistin-resistant *E. coli* and may act as underestimated reservoirs of the *mcr* genes. However, these findings should be interpreted with caution due to the small number of scientific papers identified for inclusion. Plant-based products could also represent a challenge for consumers, considering they may be ingested without any prior processing or preservation treatment. Moreover, the recommended daily intake of these products, vegetarianism and the demand for these raw and minimally processed plant-based products have increased [80]. In this sense, we emphasize the importance of carrying out more studies that clarify the epidemiology of colistin-resistant *E. coli* in these foods throughout the food chain.

In recent years, the epidemiology of colistin-resistant *E. coli* and the *mcr* genes is a topic that has aroused interest in the scientific community and in political decision-makers; therefore, studies that integrate this available information are needed. In this study, it was possible to integrate several scientific papers on the prevalence of colistin resistance in different geographic regions, foods and food-producing animals, and points of the food chain.

The evidence generated in this study allows gaining insight into the prevalence of colistin-resistant *E. coli* and the main associated *mcr* genes during the period analyzed. As was previously mentioned, global prevalence probably decreases, as demonstrated by some of the studies carried out [19,[67], [68], [69]], as more countries implement policies restricting the use of colistin in livestock, the controls and monitoring of the use of this antibiotic are optimized, and more epidemiologic studies are carried out in different regions of the world. In a “One Health” context, these results may be used as a scientific basis for the design and implementation of risk management measures in order to ensure food safety and public health, to curb the emergence and spread of antimicrobial-resistant bacteria, and to preserve the efficacy of antibiotics for future generations.

### 4.1 Limitations

There are some limitations in this study. Firstly, for different reasons, some studies did not report the requested data for inclusion in the analyses proposed in this study (A, B, and/or C). Therefore, these studies could not be included in the evaluation of prevalence. Secondly, the uneven distribution of publications around the world and the limited number of studies on colistin-resistance in some foods and food-producing animals may impact the findings in the present study. Finally, subgroup analyses to detect the sources of heterogeneity, publication bias, and heterogeneity must all be taken into account when interpreting the results reported here.

## 5 Conclusion

The results presented here showed a low prevalence of colistin-resistant *E. coli* in foods. In addition, colistin resistance was mediated primarily by the *mcr-1* gene. This fact is a public health concern because these foods can be vehicles of resistant pathogen transmission to humans or of commensal bacteria capable of transferring resistance determinants to other potential pathogens.

On the other hand, the prevalence seems to be greater in those geographic regions and intensively‐produced farm animals where colistin has been used for long periods. However, the problem has spread to other foods and food-producing animals, parts of the world, and to the main links of the food chain, probably due to anthropogenic activities and the horizontal spread of resistance-genes. Although several factors must be considered when asserting the risk that these resistant bacteria represent for the consumer (presence of virulence determinants, plasmid resistance mechanisms, adequate infective doses), their situation must be monitored.

Worldwide policies have been implemented that restrict the use of colistin in livestock; however, the use of colistin has not been completely abolished at present. Therefore, the long-term impact of these policies needs to be assessed. In addition, future studies should be conducted to evaluate the epidemiology of these bacteria in foods under minimal processing, such as fruits and vegetables, in order to determine the associated risk.

This study helps to understand the epidemiology of colistin-resistant *E. coli* and their mobile colistin resistance determinants in foods and food-producing animals through the food chain. These results may be used as a scientific basis for risk management measures under the “One Health” approach. Intensive systems of animal production, the application of good manufacturing practices during post-primary processing (in slaughterhouses and food processing plants), and correct handling and hygiene by final consumers are critical points to be reviewed in order to guarantee high standards of food safety and the preservation of consumer and population health while avoiding higher costs in the future.

## Data availability statement

The database formed and/or analyzed during this study is freely available in the Institutional Repository of the “Consejo Nacional de Investigaciones Científicas y Técnicas” (CONICET), which is our place of development of scientific activities [86]. Available on: http://hdl.handle.net/11336/184926.

## Funding sources

This study was supported by 10.13039/501100005746Universidad Nacional del Litoral (Project CAIDO 21820210100071LI).

## CRediT authorship contribution statement

**Florencia Aylen Lencina:** Writing – review & editing, Writing – original draft, Methodology, Conceptualization. **Matías Bertona:** Methodology. **María Angeles Stegmayer:** Methodology. **Carolina Raquel Olivero:** Supervision. **Laureano Sebastián Frizzo:** Writing – review & editing, Resources, Data curation. **Jorge Alberto Zimmermann:** Methodology. **Marcelo Lisandro Signorini:** Writing – review & editing, Writing – original draft, Formal analysis, Data curation, Conceptualization. **Lorena Paola Soto:** Validation, Resources. **María Virginia Zbrun:** Writing – review & editing, Writing – original draft, Resources, Investigation, Conceptualization.

## Declaration of competing interest

The authors declare that they have no known competing financial interests or personal relationships that could have appeared to influence the work reported in this paper.

## Abbreviations

*E. coli*: *Escherichia coli*
*mcr*: *mobile colistin resistance*
AMR: antimicrobial resistance
WHO: World Health Organization
*p*-estimate: pooled prevalence estimate
CI: confidence interval
